# Age-Related Exosomal and Endogenous Expression Patterns of miR-1, miR-133a, miR-133b, and miR-206 in Skeletal Muscles

**DOI:** 10.3389/fphys.2021.708278

**Published:** 2021-11-18

**Authors:** Chrystalla Mytidou, Andrie Koutsoulidou, Margarita Zachariou, Marianna Prokopi, Konstantinos Kapnisis, George M. Spyrou, Andreas Anayiotos, Leonidas A. Phylactou

**Affiliations:** ^1^Department of Molecular Genetics, Function and Therapy, The Cyprus Institute of Neurology and Genetics, Nicosia, Cyprus; ^2^Cyprus School of Molecular Medicine, The Cyprus Institute of Neurology and Genetics, Nicosia, Cyprus; ^3^Bioinformatics Department, The Cyprus Institute of Neurology and Genetics, Nicosia, Cyprus; ^4^Theramir Ltd., Limassol, Cyprus; ^5^Department of Mechanical Engineering and Materials Science and Engineering, Cyprus University of Technology, Limassol, Cyprus

**Keywords:** skeletal muscle, muscle endogenous, muscle-derived exosomes, age, myomiRs, differential expression, muscle growth

## Abstract

Skeletal muscle growth and maintenance depend on two tightly regulated processes, myogenesis and muscle regeneration. Both processes involve a series of crucial regulatory molecules including muscle-specific microRNAs, known as myomiRs. We recently showed that four myomiRs, miR-1, miR-133a, miR-133b, and miR-206, are encapsulated within muscle-derived exosomes and participate in local skeletal muscle communication. Although these four myomiRs have been extensively studied for their function in muscles, no information exists regarding their endogenous and exosomal levels across age. Here we aimed to identify any age-related changes in the endogenous and muscle-derived exosomal myomiR levels during acute skeletal muscle growth. The four endogenous and muscle-derived myomiRs were investigated in five skeletal muscles (extensor digitorum longus, soleus, tibialis anterior, gastrocnemius, and quadriceps) of 2-week–1-year-old wild-type male mice. The expression of miR-1, miR-133a, and miR-133b was found to increase rapidly until adolescence in all skeletal muscles, whereas during adulthood it remained relatively stable. By contrast, endogenous miR-206 levels were observed to decrease with age in all muscles, except for soleus. Differential expression of the four myomiRs is also inversely reflected on the production of two protein targets; serum response factor and connexin 43. Muscle-derived exosomal miR-1, miR-133a, and miR-133b levels were found to increase until the early adolescence, before reaching a plateau phase. Soleus was found to be the only skeletal muscle to release exosomes enriched in miR-206. In this study, we showed for the first time an in-depth longitudinal analysis of the endogenous and exosomal levels of the four myomiRs during skeletal muscle development. We showed that the endogenous expression and extracellular secretion of the four myomiRs are associated to the function and size of skeletal muscles as the mice age. Overall, our findings provide new insights for the myomiRs’ significant role in the first year of life in mice.

## Introduction

Skeletal muscle is one of the most abundant tissue in the body that is necessary for the locomotion and it is vital for the energy metabolism of the body (Rivas and Fielding, 2012). Skeletal muscles go through major changes such as gaining strength and mass, during the developmental stages of life, especially before adulthood (Rivas and Fielding, 2012). In the elderly population, skeletal muscle tissues are characterized by sarcopenia, an involuntary loss of skeletal muscle mass and strength (Drummond et al., 2011; Walston, 2012; Fry et al., 2015). Maintenance of skeletal muscles throughout life is essentially driven by adult myogenesis and muscle regeneration, which are both regulated by various transcription factors, such as the myogenic regulatory factors (MRFs) (Sabourin and Rudnicki, 2000; Conerly et al., 2016; Hernández-Hernández et al., 2017). MRFs are responsible to orchestrate in a timely manner the proliferation, differentiation, death, and fusion of myogenic progenitor cells that commit to the myogenic lineage, in order to form mature myofibers. Other regulatory molecules, including microRNAs (miRNAs), have been determined to play a crucial role in skeletal muscle development by either promoting or inhibiting the process of myogenesis (Parker et al., 2003; Buckingham and Rigby, 2014; Koutalianos et al., 2015).

miRNAs are endogenous small non-coding RNA molecules that repress gene expression by targeting the 3′ untranslated region (3′UTR) of their target mRNAs, leading to either inhibition of protein translation or degradation of their target mRNA (Hammond et al., 2000; Bartel, 2004). Even though many miRNAs are expressed ubiquitously in cells, some miRNAs have been characterized by tissue-specificity (Lagos-Quintana et al., 2002; Sempere et al., 2004; Guo et al., 2014). miRNAs that are predominately expressed in skeletal muscle tissue and regulate skeletal muscle function and homeostasis have been reported, including miR-1, miR-133a, miR-133b, miR-206, miR-208a, miR-208b, miR-486, miR-499a, and miR-499b, also known as myomiRs (Sempere et al., 2004; Townley-Tilson et al., 2010; Horak et al., 2016). Even though some of the myomiRs can be detected in non-muscle cells and tissues, their main function is confined to skeletal muscles. The four myomiRs, miR-1, miR-133a, miR-133b, and miR-206, have been extensively studied for their contribution in muscle development and function during embryogenesis and adult life (Koutsoulidou et al., 2011; Nohata et al., 2012; Horak et al., 2016). MRFs and other muscle associated transcriptional factors participate in the regulation of myomiR expression to ensure proper skeletal muscle development across time (Rosenberg et al., 2006; Liu et al., 2007; Sweetman et al., 2008). In addition, serum response factor (SRF) and connexin 43 (Cx43), which participate in skeletal muscle differentiation, have been identified, amongst others, as targets of the four myomiRs. Specifically, miR-133a and miR-133b regulate the expression of SRF and therefore modulate myoblast proliferation (Chen et al., 2006; Zhong et al., 2021). miR-206 and to a lesser extent miR-1 control Cx43 synthesis, which is involved in the innervation of myofibers (Anderson et al., 2006; Kim et al., 2006). Notably, the four myomiRs were reported to be elevated in the serum of patients with two muscular dystrophies, Myotonic Dystrophy type 1 (DM1) and Duchenne Muscular Dystrophy (DMD), compared to healthy individuals, indicating that they are possibly associated with the underlying mechanisms of these muscle diseases (Cacchiarelli et al., 2011; Zaharieva et al., 2013; Koutsoulidou et al., 2017). It has been also suggested that muscular dystrophies and muscle aging share common characteristics, implying that by understanding the molecular mechanisms of aging, important information will be revealed regarding the mechanisms underlying the pathogenesis of muscular dystrophies (Vinciguerra et al., 2010; Mateos-Aierdi et al., 2015).

Profiling the expression levels of the four myomiRs in skeletal muscle tissue during the first year of life in mice is poorly studied. Previous studies reported that miRNAs alter cell cycle regulation and myogenic factors’ expression, thereby affecting cellular proliferation in skeletal muscles of aged humans and mice (Drummond et al., 2011; Smith-Vikos and Slack, 2012; McGregor et al., 2014). Interestingly, while the expression levels of other miRNAs were found to change in aged skeletal muscles compared to younger samples, no significant alteration was observed in the expression levels of the four myomiRs in the examined tissues (Hamrick et al., 2010; Drummond et al., 2011; Mercken et al., 2013; McGregor et al., 2014). Furthermore, studies that aimed to investigate old and young muscles used only a few time-points during muscle growth; thus failing to give new information regarding the gradual myomiR expression levels across age (Williams et al., 2009; Hamrick et al., 2010; Di Filippo et al., 2016; Vechetti et al., 2019). More recently, we showed that these four myomiRs are encapsulated within small extracellular vesicles, called exosomes, secreted directly from skeletal muscle tissues of adolescent wild-type mice (Mytidou et al., 2021). Importantly, exosomes encapsulating the four myomiRs were found to be involved in the communication of neighboring skeletal muscle tissues of wild-type mice, suggesting a novel transfer mechanism of molecular information through the exosome route (Mytidou et al., 2021).

Exosomes are a subgroup of membranous nanovesicles with diameter of 50–150 nm, which are secreted from different cells and tissues into the extracellular space (Simons and Raposo, 2009; Raposo and Stoorvogel, 2013; Romancino et al., 2013). They have been isolated from several biofluids, like serum, urine, saliva, and cerebral spinal fluid (Gallo et al., 2012; Russo et al., 2012; Katayama et al., 2019). The encapsulated molecular repertoire within exosomes consists of proteins, lipids and several RNA molecules, including miRNAs (Zhang et al., 2016; De Gasperi et al., 2017; Li et al., 2018). Exosomes are released under both normal and pathological conditions reflecting the status of the producing cell or tissue. Exosomal cargo is therefore, directly associated to the exosome-releasing cells, attributing their functional impact to their cell origin and content heterogeneity (De Gasperi et al., 2017; Steinbichler et al., 2017). Several scientific reports have emphasized on the important role of exosomes in intercellular communication, primarily in malignant and immune response cell culture systems (Delcayre et al., 2005; Grange et al., 2014; Lima and Möller, 2018). Very few studies, however, have focused on exosomes and their content released from skeletal muscles. In particular, published reports examined the secretion of exosomes from muscle cell lines, isolated muscle fibers, skeletal muscle tissues cut into pieces and intact skeletal muscles (Forterre et al., 2014; Jalabert et al., 2016; Nie et al., 2019; Mytidou et al., 2021). Although, extensive studies have been performed regarding the four myomiRs in skeletal muscle tissues, their endogenous expression levels and exosomal release from skeletal muscles during the first year of mice life have not been examined yet. An in-depth investigation of the levels of the four myomiRs at different age time-points of mice will provide novel evidence for their role in skeletal muscle tissue.

The aim of this study was therefore, to investigate the endogenous levels of miR-1, miR-133a, miR-133b, and miR-206, in five different skeletal muscles located on the hindlimbs (extensor digitorium longitus (EDL), soleus, tibialis anterior (TA), gastrocnemius and quadriceps) and their corresponding muscle-derived exosomal levels, during the first year of life in wild-type mice. Initially, we generated an endogenous and a muscle-derived exosomal myomiR profile for each skeletal muscle, starting from the pre-adolescent age (2 weeks) until adulthood of wild-type mice (52 weeks). Endogenous miR-1, miR-133a, and miR-133b levels were found to increase across age, showing a positive correlation with muscle growth for all five skeletal muscles. Endogenous miR-206, on the contrary, was found to exhibit a muscle fiber type specificity for all ages. Moreover, the differential expression of the four myomiRs in skeletal muscles was shown to be inversely correlated to the SRF and Cx43 synthesis. Furthermore, we showed that muscle-derived exosomal myomiR levels increased until early adolescent phase. Finally, our findings revealed that up to early adolescence, murine skeletal muscles express high myomiR levels and, at the same time, limit their exosomal release compared to the older ages, emphasizing the myomiRs’ significance during the period of rapid muscle growth. Our data provide new information on the mechanisms underlying the growth of skeletal muscle during the first year of life in mice, which would be useful for future studies regarding skeletal muscle tissue function in health and disease.

## Materials and Methods

### Ethics Statement

All animal studies were performed following protocols approved by the Cyprus Legislation for the protection and welfare of animals, Laws 1994–2013.

### Experimental Animals and Tissues

Animals were housed and handled in a temperature- and humidity-controlled room under pathogen-free standard conditions on a regular 12 light/12 dark cycle. Male mice on a C57BL/6 background aged from 2 to 52 weeks were used for profiling the four myomiRs. Blood samples were collected from the orbital sinus under general anesthesia, prior to the mice sacrifice via cervical dislocation. Five skeletal muscles located on the hind limb, EDL, soleus, TA, gastrocnemius, and quadriceps, were extracted and any fat tissue was carefully removed without further injuring the muscle tissues. Gastrocnemius and quadriceps are large muscle groups that consist of two and four individual muscles, respectively, and all parts were collected for our analysis. Body and muscle weights were recorded for all mice.

### Exosome Isolation From Tissues

Fresh skeletal muscle tissues were collected from mice and immediately placed in vials containing Dulbecco’s Modified Eagle’s Medium (DMEM; Invitrogen) supplemented with 10% Exosome-Depleted Fetal Bovine Serum (Exo-FBS; System Biosciences), 2% GlutaMAX (Invitrogen), and 1% Penicillin-Streptomycin (P-S; Invitrogen). The tissues were incubated in a humidified incubator set at 37°C and 5% CO_2_ for 24 h, allowing them to release extracellular vesicles, including muscle-derived exosomes, under conditions resembling the natural environment within the body. Medium was then collected and filtrated through 0.22 μm filters (Millipore) to remove cell debris and extracellular vesicles of a larger size. ExoQuick-TC^TM^ Exosome Precipitation Solution (System Biosciences) was used to precipitate muscle-derived exosomes from the medium, according to manufacturer’s instructions. ExoQuick^TM^ Exosome Precipitation Solution (System Biosciences) was used to isolate exosomes from serum samples following manufacturer’s instructions. In brief, appropriate amount of ExoQuick-TC^TM^ or ExoQuick^TM^ reagent was added to the filtered medium or serum samples, respectively, and incubated overnight at 4°C. The next day, the mixtures were centrifuged at 1,500 × g for 30 min and the exosome-containing pellet was collected and dissolved in the proper solution for further analysis.

### Scanning Electron Microscopy

Exosomes isolated from serum and skeletal muscles of 16-week-old male mice were subjected to fixation using 4% paraformaldehyde. Samples were mounted on aluminum specimen stubs and sputtered using gold/palladium (Au/Pd). High resolution scanning electron microscopic analysis was carried out at 30.00 kV in a FEI Quanta 200 microscope and images were processed using the MountainsMap SEM Topo Version 7.3 software.

### Tunable Resistive Pulse Sensing Analysis

The concentration and size distribution of the isolated serum and muscle-derived exosomes from 16-week-old male mice were examined by Tunable Resistive Pulse Sensing (TRPS), using the qNano gold instrument (IZON Science). The instrument was set up and calibrated following the manufacturer’s recommendations. The nanopore membrane NP200 was used and was axially stretched to 47.01 mm. The apparatus for both calibration and sample measurements was operated at a voltage of 0.38 V and a pressure equivalent to 20 mbar. The exosomes isolated from serum was 10-fold diluted whereas exosomes derived from muscle tissues was 20-fold diluted. All measurements were calibrated with 210 nm polystyrene beads that were appropriately diluted (1:1,000) (IZON Science). As particles move through the membrane, they cause a change in the ionic current, which was recorded and analyzed using the Izon Control Suite software v3.3 (IZON Science).

### Protein Extraction and Western Blot Analysis

Skeletal muscle tissues were homogenized and exosomes were lysed in lysis buffer consisting of 150 mM NaCl, 10 mM Tris-HCl pH = 7.6, 10% Glycerol, 0.5% Tween, 10 mM Mercaptoethanol and supplemented with 2X EDTA-free Protease Inhibitor cocktail. The lysates were left on ice for at least 1 h. Tissue and exosomal lysates were sonicated six and three times, respectively, with 30 s on ice in-between treatments, followed by centrifugation for 30 min at 4°C. The supernatant was collected and stored in −80°C for future use. 20–40 μg of protein extracts were separated on 10–12% SDS polyacrylamide gels. Proteins were, next, transferred to PVDF membranes, followed by blocking in 3–6% no-fat milk for 30 min–1 h. Membranes were incubated overnight with anti-GAPDH (mouse monoclonal, 1:3,000 dilution; Santa Cruz Biotechnology), anti-SRF (mouse monoclonal, 2:750 dilution; Santa Cruz Biotechnology), anti-Connexin43 (rabbit polyclonal, 2:750 dilution; Santa Cruz Biotechnology), anti-CD63 (mouse monoclonal, 1:500 dilution; Abcam), anti-CD81 (mouse monoclonal, 1:750 dilution; Abcam), anti-TSG101 (mouse monoclonal, 1:2,000 dilution; Santa Cruz Biotechnology), and anti-Calnexin (rabbit monoclonal, 1:3,000 dilution; Abcam) primary antibodies. The next day, membranes were washed thrice and incubated with anti-mouse IgG or anti-rabbit IgG secondary antibodies conjugated to HRP (1:5,000 dilution; Santa Cruz Biotechnology) for 2 h. Proteins were visualized using UVP BioSpectrum^®^ 810 Imaging System or Vilber Fusion Solo S. To determine changes in protein abundances in skeletal muscle tissues across time, SRF and Cx43 concentration levels were normalized over the internal GAPDH control. GelAnalyzer software was used to evaluate band intensity.

### microRNA Analysis

Fresh skeletal muscles were homogenized, employing the Precellys^®^ 24 tissue homogenizer, and total RNA, enriched in miRNAs, was extracted using the mirVana^TM^ miRNA Isolation Kit (Invitrogen), according to manufacturer’s instructions. RNA was also extracted from exosomes using the Total Exosome RNA and Protein Isolation Kit (Invitrogen), following the manufacturer’s instructions. Reverse transcription of 10 ng from the RNA samples followed, using the TaqMan MicroRNA Reverse Transcription Kit (Applied Biosystems) according to the manufacturer’s instructions. Real-time PCR amplification was carried out with TaqMan MicroRNA Assays (Applied Biosystems) specific for miR-1, miR-133a, miR-133b, and miR-206 using the QuantStudio^TM^ 7 Flex System (Applied Biosystems). Tissue and exosomal miRNA abundances were normalized to the endogenous control snoR-135 or spike-in control cel-miR-39, respectively.

### Data and Statistical Analysis

Relative quantification (RQ) of miRNA levels was calculated using the equation *RQ* = 2^−(*Ct*_*control*_−*Ct*_*miRNA*_)^, where Ct corresponds to the threshold cycle obtained by the QuantStudio^TM^ 7 Flex System Software (Applied Biosystems). Means and standard error of the mean (SEM) were calculated from three independent experiments (*n* = 3). For further analysis of our results, the fold change (FC) of the RQ values over the 2-week values was determined for each miRNA. Moreover, normalized RQ values with respect to the weight of each skeletal muscle for all ages separately were determined by calculating the ratio of the RQ miRNA levels over muscle weight. Statistical significance was assessed for the correlation analysis of the data and was set to less than 0.05 (*p* < 0.05).

### Correlation Analysis

Correlation analysis was performed for the endogenous, muscle-derived and serum miR-1, miR-133a, miR-133b, and miR-206 abundances across time using the packages corrplot (v0.84), ggplot2 (v3.3.5), gplots (v.3.1.1), Hmisc (v.4.5.0), RColorBrewer (v.1.1.2) in R language (4.0.2) in R studio (R Core Team, 2013; Neuwirth, 2014; Wickham, 2016; Wei and Simko, 2017; Warnes et al., 2020; Harrell, 2021). We performed normality tests for all time-series using the Shapiro-Wilk’s test, a non-parametric test (recommended for less than 50 samples), which evaluates whether the data distribution is significantly different from the normal distribution (for *p* < 0.05) (Mishra et al., 2019). Since not all data distributions were found to have a *p*-value greater than 0.05 (which would imply that the data distribution was not significantly different from the normal distribution), the Spearman correlation for testing for pairwise correlations across different data distributions was used, as it is more appropriate for non-normal distributions. The Spearman correlation rho method calculates the correlation between the rank of two variables × (myomiR expression levels) and y (age). A rho value of −1 denotes a strong negative correlation, a value 0 indicates that there is no association between × and y and a value of 1 means that there is a strong positive correlation.

## Results

### Endogenous miR-1, miR-133a, and miR-133b Levels Increase With Age, While miR-206 Expression Is Specific Only to Soleus

**FIGURE 1:**
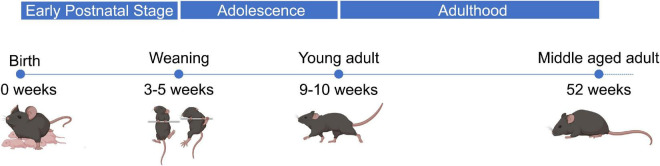
Developmental stages throughout lifetime of wild-type mice. Wild-type male mice go through several developmental stages during their lifetime. Up to 4 weeks after birth, which is the early postnatal period, the pups are completely dependent on their mother for food and protection. During this time-period the pups gain weight with an increasing rate. After 4 weeks until 9–10 weeks, male mice mature sexually and become more aggressive. Through this period, which is known as adolescence, the mice become more independent, their weight is still increasing, and they experience many behavioral changes. Finally, 10 weeks after birth wild-type male mice have fully transitioned to adulthood. Their bodies have stopped gaining weight, they are sexually active, and they mature regarding their behavior. Around 1 year after their birth, inbred mice begin to show signs of functional and behavioral decline, while the majority of them have reached the post-productive era of their life.

Extensive studies have been conducted regarding the expression and function of miR-1, miR-133a, miR-133b, and miR-206 in muscle (Townley-Tilson et al., 2010; Horak et al., 2016). The majority of published studies has focused on the expression of myomiRs and their role in the process of myogenesis during early development or in aged humans and mice (Koutsoulidou et al., 2011; Di Filippo et al., 2016; Vechetti et al., 2019). Currently, there is little information about their expression and regulatory activity during the life span of mice. The period from birth until 4 weeks of life is considered to be the early postnatal phase of mice during which the pups mostly depend on their mother and rapidly develop behavioral and motor abilities. The period from 4 to 9 weeks after birth represents the phase of adolescence during which mice are fully independent, more aggressive, and sexually mature. 9–10 weeks after birth, mice are fully transitioned to adulthood, characterized by behavioral maturity, and have a fully developed body. One year after birth mice enter the post-reproductive period experiencing a decline in their functionality and behavioral performance. Finally, 2 years after birth the decline is more apparent than before and the mice are close to the end of their lifespan (Figure 1; Lhotellier and Cohen-Salmon, 1991; Yuan et al., 2011; Brust et al., 2015).

**FIGURE 2:**
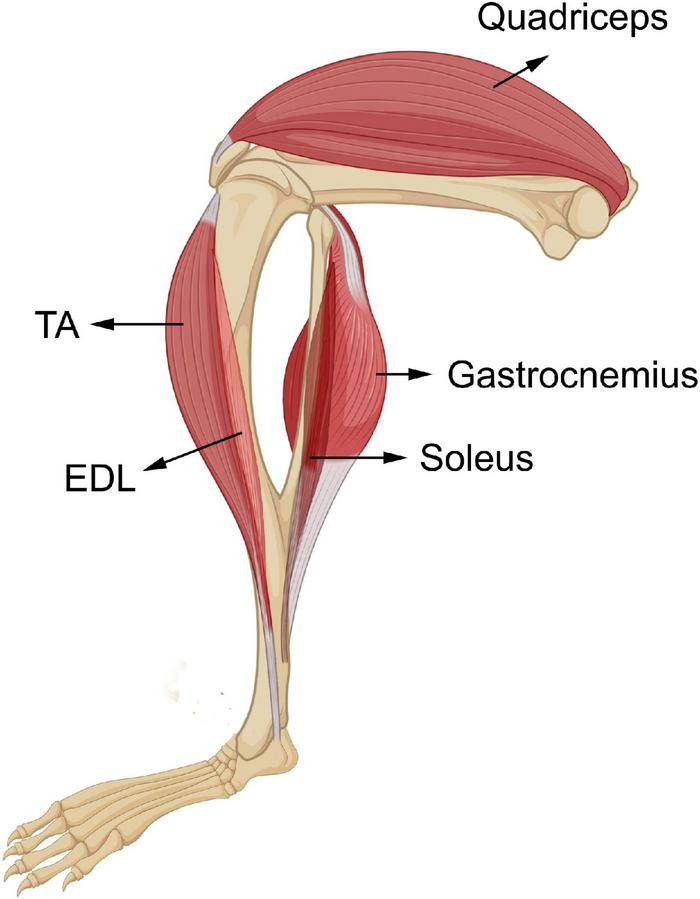
Skeletal muscles of the hindlimbs of wild-type male mice. Five skeletal muscles from the hindlimbs of wild-type mice, ELD, soleus, TA, gastrocnemius, and quadriceps were collected in this study. EDL and soleus are the two smallest skeletal muscles under investigation, TA is a medium-sized skeletal muscle, and gastrocnemius and quadriceps are the two heaviest tissues. EDL and TA are located on the front of the hindlimb underneath the knee. Soleus and gastrocnemius are located on the back lower part of the hindlimb. Their tendons are in the Achilles’s tendon. Quadriceps is one of the biggest skeletal muscles of the organism and resides on the front of the upper part of the hindlimb.

**FIGURE 3:**
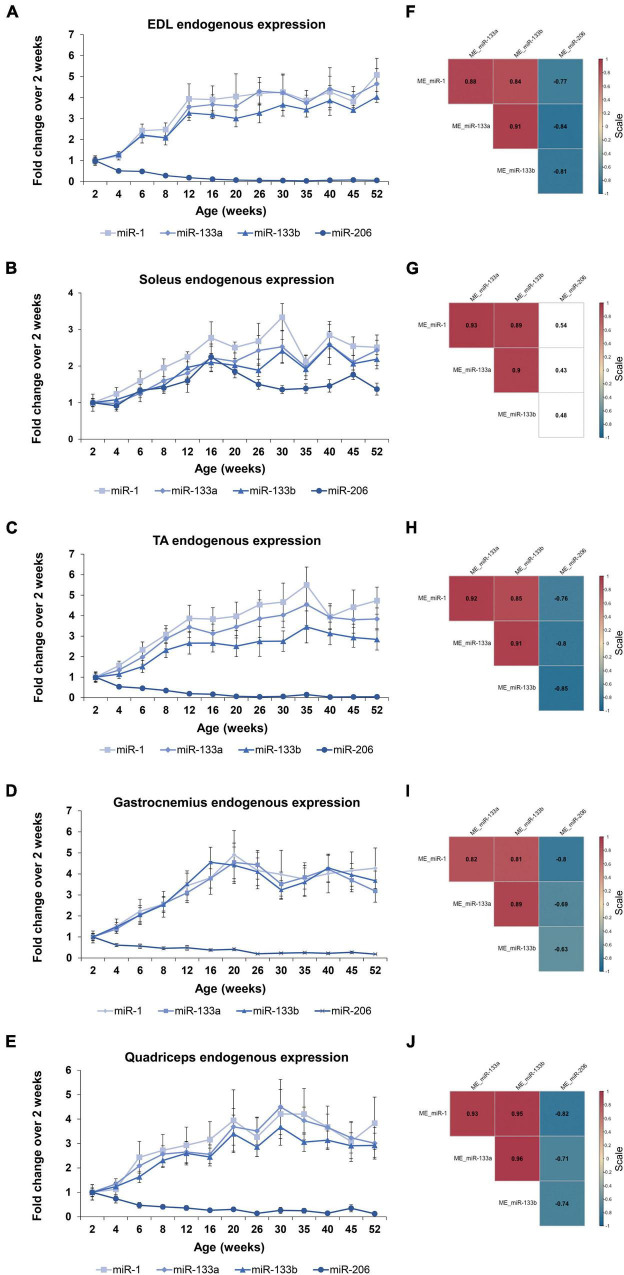
Expression profile of the endogenous myomiR levels associate to the age of the mice. Skeletal muscles from wild-type male mice of ages from 2 to 52 weeks (thirteen different time-points) were extracted and analyzed for the endogenous levels of the four myomiRs (*n* = 3). **(A–E)** Fold change (FC) graphs relative to 2 weeks levels of the endogenous myomiR levels in five skeletal muscle tissues. miR-1, miR-133a, and miR-133b relative levels were increased by 4–5 times compared to 2 weeks, until the age of 12 weeks in **(A)** EDL, **(C)** TA, **(D)** gastrocnemius, and **(E)** quadriceps, and then reached a plateau phase. miR-206 levels in the same tissues were found to be significantly decreased across age. **(B)** In soleus the endogenous FC levels of all four myomiRs were identified to be increased by 2–3 times, compared to the 2-week levels. Data on graphs represent the mean values ± SEM. **(F–J)** Correlation coefficients were calculated using the Spearman method revealing a significant positive association of the three myomiRs, miR-1, miR-133a, and miR-133b. miR-206 was negatively correlated to the other three myomiRs for all the skeletal muscles, apart from soleus. Correlation (rho) values are shown within the squares and those close to +1 (red) denote a strong positive correlation, and values close to –1 (blue) a strong negative correlation. Correlation values with no significance are in white. *p* < 0.05; ME, muscle-endogenous.

In order to determine the four myomiR expression patterns across age, five skeletal muscle tissues, EDL, soleus, TA, gastrocnemius, and quadriceps, located on the hindlimbs of wild-type male mice from 2 to 52 weeks old were analyzed for the myomiR endogenous levels (Figure 2). Initially, relative quantification of the four myomiRs was determined using snoR-135 as endogenous control (Supplementary Figure 1). Fold change analysis showed that the endogenous levels of miR-1, miR-133a, and miR-133b increase rapidly during adolescence and early adulthood (4–12 weeks), before reaching a plateau phase compared to the 2-week levels, for all skeletal muscles under investigation (Figures 3A–E). In particular, miR-1, miR-133a, and miR-133b expression levels showed a four to fivefold increase in EDL, TA, gastrocnemius, and quadriceps compared to the 2-week levels, whereas in soleus they presented an approximately two to threefold increase (Figure 3). On the contrary, the endogenous levels of miR-206 presented a gradual decrease across time relative to 2 weeks in EDL, TA, gastrocnemius, and quadriceps (Figures 3A,C–E), whereas in soleus miR-206 expression levels increased (Figure 3B). Of note, the prominent myofiber type in soleus muscle is the oxidative type and miR-206 has been reported in previous studies to be expressed in a myofiber type specific manner (Bjorkman et al., 2020; Mytidou et al., 2021).

Correlation analysis using the Spearman method verified that miR-1, miR-133a, and miR-133b endogenous expression levels follow a similar trend during the first year of life in mice, showing significant strong positive correlations between the three miRNAs (rho > 0.81; *p* < 0.05) for all five skeletal muscles (Figures 3F–J). In addition, miR-206 was found to be significantly negative correlated to the other three myomiRs across time for the glycolytic muscle tissues i.e., EDL, TA, gastrocnemius, and quadriceps (rho < −0.63; *p* < 0.05) (Figures 3F,H–J). By contrast, the relative miR-206 expression levels in soleus, relative to 2 weeks, were positively correlated with the other three myomiR endogenous levels, across the age groups (Figure 3G).

**FIGURE 4:**
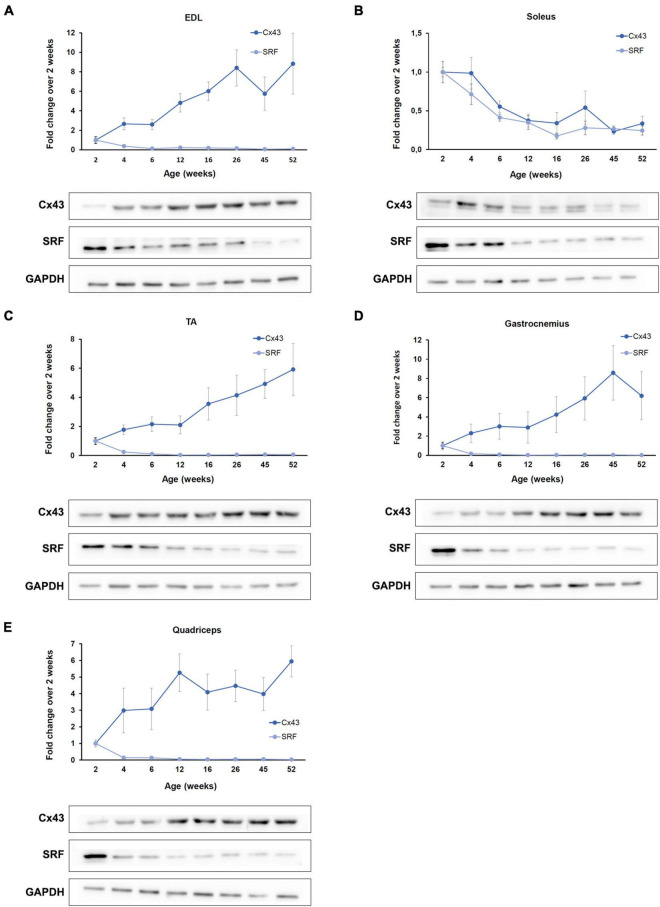
SRF and Cx43 production is inversely associated to the myomiR expression. The function of the four myomiRs in the five skeletal muscles was further explored by evaluating the production levels of two of their established targets, SRF and Cx43. **(A–E)** Fold change (FC) levels obtained from western blot analysis of SRF and Cx43 across different ages and compared the 2-week levels are shown. SRF was determined to be inversely correlated to the endogenous expression of miR-1, miR-133a, and miR-133b for all five skeletal muscles. Cx43 was found to be inversely correlated to the endogenous expression profile of miR-206. The glycolytic muscles **(A)** EDL, **(C)** TA, **(D)** gastrocnemius, and **(E)** quadriceps were shown to express increasing levels of Cx43 as the mice age, while **(B)** the oxidative soleus muscle was identified to express decreasing Cx43 levels with muscle development. Representative western blot images are shown for each skeletal muscle. GAPDH was served as normalization control in three western blot analyses (*n* = 3). Data on graphs represent the mean values ± SEM.

The four myomiRs under investigation have been established as main regulators of myogenesis at the post-transcriptional level (McCarthy, 2008; Horak et al., 2016). miR-1 and miR-206 were reported to regulate muscle cell differentiation, while miR-133a and miR-133b control muscle cell proliferation (Horak et al., 2016). Their synergistic functions promote healthy myogenesis resulting in the formation of new muscle fibers thus increasing muscle mass. To further complement the myomiR differential expression analysis in murine skeletal muscles during the acute developmental changes in the first year of life, we investigated their effect on the production of two validated targets, SRF and Cx43 in the same tissues. Both proteins have been identified to be involved in different stages of myogenesis and their absence hinders normal muscle development and homeostasis (Soulez et al., 1996; Araya et al., 2003; Li et al., 2005). SRF is an established target of miR-133 and western blot analysis revealed that its expression profile is inversely correlated to the miR-133a and miR-133b endogenous patterns across the first year of muscle growth in all skeletal muscles under investigation (Figure 4). Specifically, SRF levels were determined to decrease to minimal levels compared to the 2-week values, as mice age and grow. Cx43 synthesis, however, which is regulated mainly by the function of miR-206 and to a lesser extend miR-1, was identified to follow a different pattern with respect to the two types of muscle tissues studied in this work i.e., the oxidative and glycolytic muscles (Figure 4). Cx43 protein levels were shown to be inversely associated to the expression levels of miR-206; essentially, Cx43 was found to decrease in the oxidative muscle soleus and increase in the glycolytic muscles EDL, TA, gastrocnemius, and quadriceps, during muscle growth.

**FIGURE 5:**
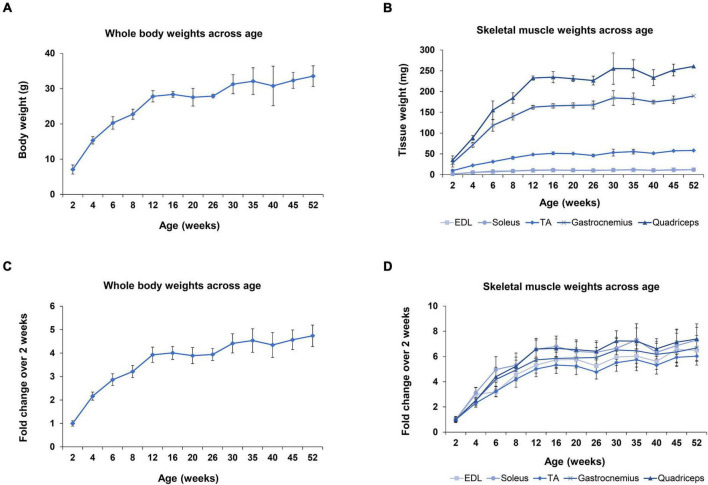
Whole body and skeletal muscle weights during the first year of life in wild-type mice. Whole body and skeletal muscle tissues weight were recorded for all mice under investigation (*n* = 3). **(A)** Body weights of mice increased rapidly until 12 weeks of age, which corresponds to the period that mice switch from adolescence to adulthood. **(B)** EDL and soleus muscles are the lightest tissues examined in this project with similar weights across all ages. TA muscle is approximately 5 times heavier than EDL and soleus. Gastrocnemius and quadriceps muscles are the heaviest tissues, weighing 18–20 times more than the smaller tissues, EDL and soleus, for all the examined ages. **(C)** Fold change of mice body weights compared to the 2-week weight demonstrated a fourfold increase until the age of 12 weeks and then reached a plateau. **(D)** Fold change of the five skeletal muscle weights compared to the 2-week time-point showed the same trendline as the body weight profile in C, but with larger fold changes. All tissues reached a plateau phase at 12 weeks and displayed a five to sevenfold increase. Data on graphs illustrate the mean values ± SEM.

**FIGURE 6:**
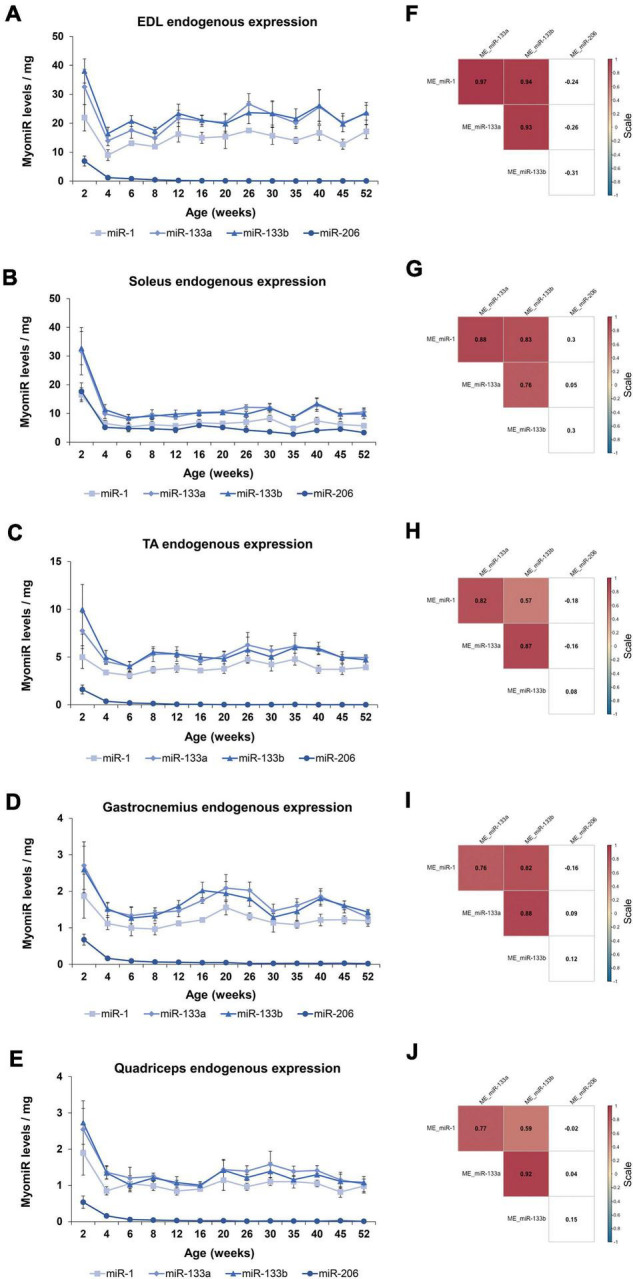
Endogenous expression levels of the four myomiRs associate to the skeletal muscle growth. The endogenous myomiR levels were further normalized to the skeletal muscle weights across all the ages. **(A–E)** At the 2-week time-point (early postnatal stage) the normalized endogenous myomiR levels were found to be increased for all the skeletal muscles. During adolescence and adulthood their endogenous levels were identified to be decreased and remained at a constant low level. **(A)** EDL tissue was found to express the highest amount of myomiR levels per muscle weight compared to the rest of the tissues. The two heaviest tissues, **(D)** gastrocnemius and **(E)** quadriceps, were determined to express the lowest myomiR levels per muscle weight. Normalized endogenous miR-206 abundance remained at high levels in **(B)** soleus for all the ages. Data on illustrations represent the mean values ± SEM. **(F–J)** Heatmaps of the correlation coefficients were generated using the Spearman method. The three myomiRs, miR-1, miR-133a, and miR-133b showed significantly positive correlations between them for all the skeletal muscles, whereas miR-206 was uncorrelated with the other three myomiRs. Correlation (rho) values are shown within the squares and those close to +1 (red) denote a strong positive correlation, and values close to –1 (blue) a strong negative correlation. Correlation values with no significance are in white. *p* < 0.05; ME: muscle-endogenous.

During the first year of life in mice dramatic changes are observed in their development; especially in the first 8 weeks of age, which marks the transition period of adolescence to adulthood. One of the prominent developmental changes occurring as the mice age is the acute muscle growth. Whole body and skeletal muscle tissue weights were recorded, in order to further analyze the myomiR expression levels based on muscle growth (Figures 5A,B). The body weight of mice was determined to increase 4 times from 2 to 12 weeks of age, and then it reached a plateau (Figure 5C). Moreover, the weights of the five skeletal muscles were found to increase 5–7 times in the first 12 weeks of age in mice, and then followed a plateau (Figure 5D). Notably, the fold change of the body and skeletal muscle weights across age relative to the 2 weeks values presented a similar trend to the fold change of the endogenous miR-1, miR-133a, and miR-133b levels compared to 2 weeks (Figures 3A–E, 5C,D). This observation indicates that muscle growth and myomiR endogenous expression across age are positively correlated i.e., when skeletal mass increases, the three myomiR endogenous levels also increase. To further determine the relation between muscle development and myomiR expression during the first year of life in mice, we also evaluated the endogenous myomiR levels normalized to the weight of each skeletal muscle across age, by dividing the myomiR endogenous levels with muscle weight. The normalized expression levels of the three myomiRs, miR-1, miR-133a, and miR-133b, were determined to be relatively stable during the entire adolescence and adulthood until the age of 52 weeks (Figures 6A–E). The myomiRs were found to be more abundant in the two smallest skeletal muscles, EDL and soleus compared to the largest muscles TA, gastrocnemius, and quadriceps when they were normalized to the corresponding muscle weight (Figures 6A,B). Of note, the two larger muscles, gastrocnemius, and quadriceps, presented the lowest myomiR levels per mg (Figures 6D,E). At 2 weeks, which is the early postnatal period, the four myomiRs show higher normalized expression levels to the muscle weights when compared to the older ages in all five skeletal muscles (Figures 6A–E). These results show that even though at 2 weeks of age the skeletal muscles are smaller in size, they express higher abundances of the four myomiRs. Further correlation analysis revealed that the three myomiRs miR-1, miR-133a, and miR-133b are significantly positive correlated between them (rho > 0.78; *p* < 0.05) for all investigated skeletal muscles (Figures 6F–J). On the contrary, miR-206 was found to be uncorrelated with any of the other three myomiRs for all muscles (Figures 6F–J).

### Secreted Exosomal MyomiR Levels From Skeletal Muscles Increase During Adolescence

**FIGURE 7:**
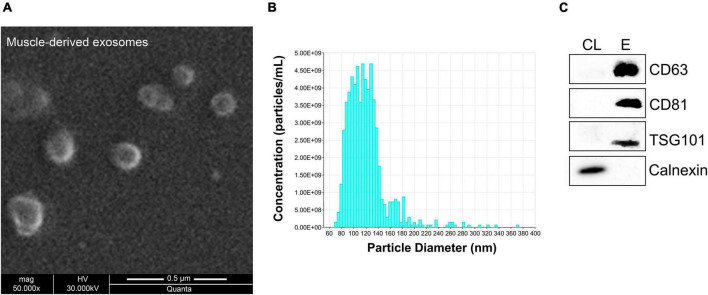
Characterization of muscle-derived exosomes. Skeletal muscles were harvested from 16-week-old wild-type mice and incubated in tissue culture medium for 24 h, allowing for the tissues to secrete exosomes in the surrounding environment. Muscle-derived exosomes were then isolated from the culture medium and characterized based on their physical and molecular properties. **(A)** Micrographs were obtained using scanning electron microscopy at 50,000× magnification illustrating spherical structures with 100–150 nm diameter. **(B)** TRPS analysis demonstrated that isolated muscle-derived exosomes have 121 nm mean diameter. **(C)** Western blot analysis was performed for the detection of positive exosomal markers CD63, CD81, and TSG101 in exosomal protein extracts (E) derived from skeletal muscles compared to cell protein lysates (CL). Calnexin was served as negative control.

**FIGURE 8:**
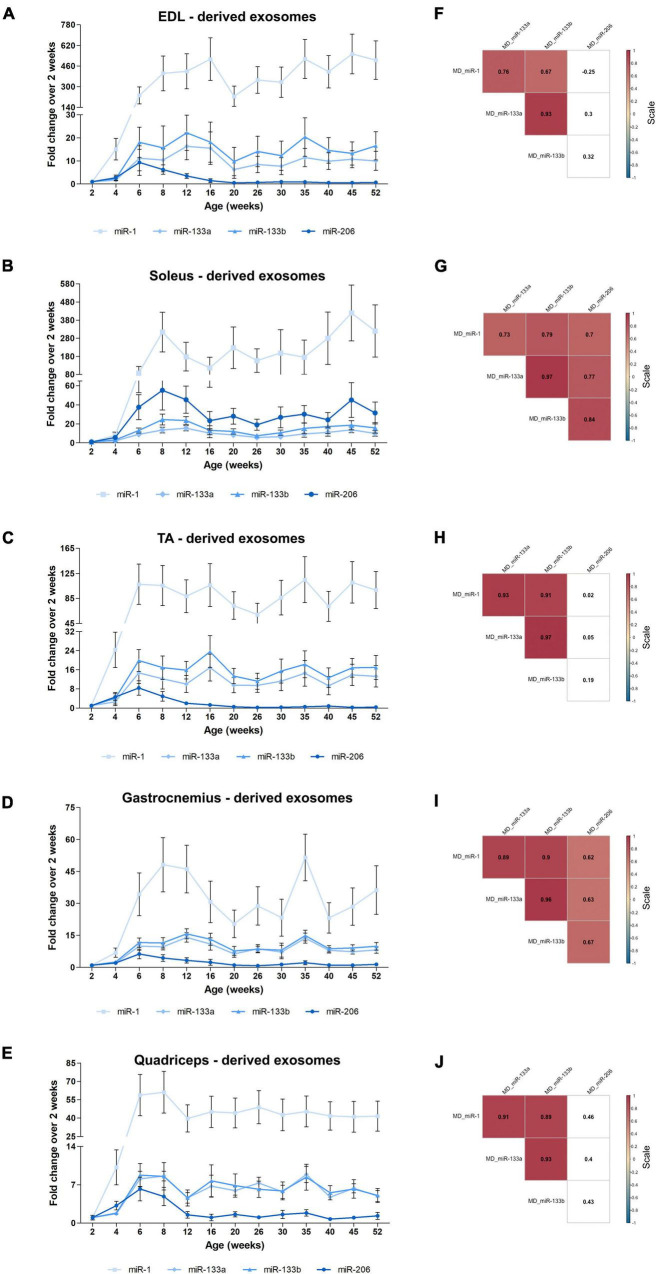
Muscle-derived exosomal myomiR levels correlate with the age of the mice. Exosomes isolated from EDL, soleus, TA, gastrocnemius, and quadriceps of wild-type mice were analyzed for their encapsulated myomiR levels (*n* = 3). **(A–E)** Fold changes of the exosomal myomiR levels relative to the 2-week levels were calculated and are illustrated across age. During the first 4 weeks of life (early postnatal stage) the five skeletal muscles secreted low levels of exosomal myomiRs, which then increased rapidly until the age of 6 weeks (adolescence). Muscle-derived exosomal miR-1 presented big fluctuations relative to the 2-week age time-point for all five skeletal muscle tissues. miR-1, miR-133a, and miR-133b remained at a constant level after the 6 weeks age compared to 2 weeks. miR-206 levels were found to decrease with age for the glycolytic muscles **(A)** EDL, **(C)** TA, **(D)** gastrocnemius, and **(F)** quadriceps, whereas in **(B)** soleus miR-206 levels remained high. **(E)** Quadriceps which is the heaviest muscle tissue demonstrated the lowest degree of fold change of exosomal myomiRs when compared to the 2-week levels among the examined skeletal muscles. Data on graphs represent the mean values ± SEM. **(F–J)** Heatmaps generated by the Spearman correlation coefficients revealed that secreted exosomal miR-1, miR-133a, and miR-133b are positively correlated for all skeletal muscles. miR-206 was found to be positively correlated to the other three myomiRs only in soleus-derived exosomes. Correlation (rho) values are highlighted within the squares and those close to +1 (red) denote a strong positive correlation, and values close to –1 (blue) a strong negative correlation. Correlation values with no significance are in white. *p* < 0.05; MD: muscle-derived.

Recently, strong emphasis has been placed on exosomes and their functional impact, especially in skeletal muscle tissues (Chen et al., 2017; Nie et al., 2019; Rome et al., 2019). Very few studies have been conducted regarding exosomes released by skeletal muscles. Specifically, release of exosomes from pieces of skeletal muscles or isolated myofibers or murine muscle cell cultures was previously reported (Forterre et al., 2014; Jalabert et al., 2016; Rome et al., 2019). We recently showed that exosomes isolated directly from intact skeletal muscle tissues encapsulate the four myomiRs (Mytidou et al., 2021). To further investigate the continuous skeletal muscle secretion profile of the four myomiRs across age, muscle-derived extracellular vesicles, including exosomes, were collected from intact EDL, soleus, TA, gastrocnemius and quadriceps muscles, for thirteen different age groups, as described previously (Mytidou et al., 2021). Isolation of exosomes was confirmed by their physical and molecular characterization using scanning electron microscopy images, TRPS analysis and western blotting (Figure 7). Moreover, TRPS revealed that all skeletal muscles secrete small extracellular vesicles of similar size, in the range of exosomes (Table 1). Following that, miRNAs were isolated from muscle-derived exosomes and real-time PCR analysis showed that miR-1, miR-133a, miR-133b, and miR-206 are encapsulated within muscle-derived exosomes for all ages of the examined mice in our study (Figures 8A–E). Relative quantification of the four myomiR levels within muscle-derived exosomes was determined using the spike-in control cel-miR-39 (Supplementary Figure 2). Fold change levels of the exosomal myomiR cargo relative to 2-week myomiR levels were evaluated across age and across the five skeletal muscles. Quadriceps, which is the heaviest muscle in this study (Figure 5B), revealed the lowest fold changes of its exosomal myomiR levels across age compared to 2 weeks (Figure 8E). In addition, it was shown that, during the early postnatal stage (2–4 weeks), the five skeletal muscles secreted minimal levels of the four myomiRs (Figures 8A–E). After the first 4 weeks, muscle-derived exosomal myomiR levels were determined to increase rapidly. In particular, exosomal miR-1 levels showed the greatest rise compared to 2 weeks for all skeletal muscles; especially for the two smaller tissues, EDL and soleus (Figures 8A,B). Moreover, both miR-133a and miR-133b exosomal levels were determined to increase rapidly from the age of 4 to 6 weeks before reaching a plateau for all five skeletal muscles (Figures 8A,B). EDL, soleus, TA and gastrocnemius released similar miR-133a and miR-133b levels relative to the 2 weeks levels. By contrast, miR-206 exosomal cargo was found to increase until the age of 6 weeks and thereafter it decreased rapidly for the glycolytic muscles EDL, TA, gastrocnemius, and quadriceps (Figures 8A,C–E). Soleus-derived exosomal miR-206 levels, however, were identified to increase after the early postnatal age (4 weeks) and remained at high levels with some fluctuations for older ages (Figure 8B). Subsequent correlation analysis revealed that exosomal levels of miR-1, miR-133a, and miR-133b share a significant positive correlation between them (rho > 0.67; *p* < 0.05) when secreted from the five skeletal muscles under investigation (Figures 8F–J). Soleus-derived exosomal levels of miR-206 were also found to be significantly positive correlated (rho > 0.7; *p* < 0.05) to the other three myomiRs (Figure 8G).

**TABLE 1:** The five hindlimb skeletal muscles secrete exosomes of the same size.

	EDL	Soleus	TA	Gastrocnemius	Quadriceps
Exosomes mean diameter (nm)	155.3 ± 2.3	145.7 ± 2.4	158.3 ± 2.4	166.7 ± 2.3	137.0 ± 6.1

**FIGURE 9:**
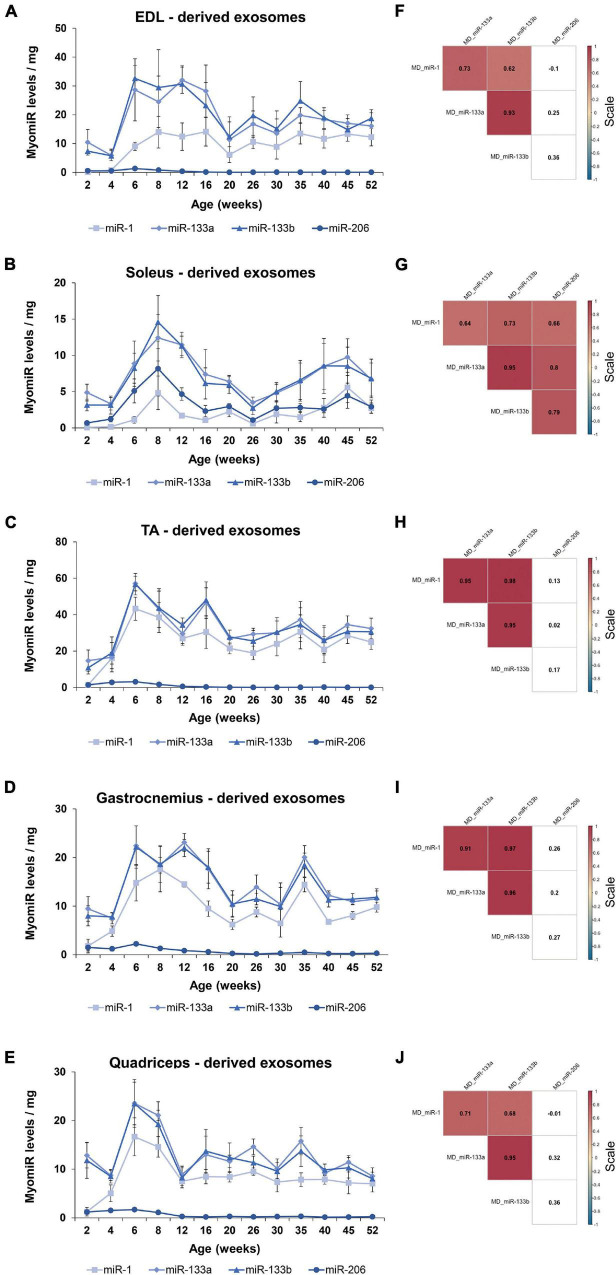
Exosomal myomiR levels normalized to the weight of the skeletal muscles follow similar profile patterns. Muscle-derived exosomal myomiR levels presented a similar correlation to the growth of muscle with age. **(A–E)** Exosomal myomiR abundances per muscle weight derived from all the muscle tissues were lower in the first 4 weeks of life compared to the older ages. During early adolescence, the exosomal myomiR levels reached a peak followed by similar trendlines for the majority of the skeletal muscles. Normalized miR-206 levels encapsulated within exosomes derived from all the skeletal muscle tissues were very low throughout the first-year lifespan **(A,C–E)**, with the exception of soleus-derived exosomes in which the levels of miR-206 were higher **(B)**. Data on illustrations represent the mean values ± SEM. **(F–J)** Correlation analysis with the Spearman method revealed a significant positive correlation between the secreted miR-1, miR-133a, and miR-133b exosomal levels normalized to muscle weights for all the skeletal muscles. **(G)** A significant positive correlation was demonstrated between miR-206 and the other three myomiRs in soleus-derived exosomal levels normalized to the soleus weight. Correlation (rho) values are shown within the squares and those close to +1 (red) denote a strong positive correlation, and values close to –1 (blue) a strong negative correlation. Correlation values with no significance are in white. *p* < 0.05; MD: muscle-derived.

Following the analysis of the four myomiR exosomal levels released by the five skeletal muscles, their association to the tissues’ weights was further examined. Normalization of the exosomal myomiR abundance over muscle weights revealed a similar pattern for all five muscles (Figures 9A–E). In particular, during early postnatal stage (up to 4 weeks) the five muscle tissues were found to release low exosomal myomiR levels normalized to the corresponding tissue weight, while in adolescence, a rapid increase was observed in the myomiR exosomal levels. During adulthood muscle-derived exosomal miR-1, miR-133a, miR-133b abundances per muscle weight followed similar trends for all skeletal muscles under investigation (Figures 9A–E). miR-206 levels encapsulated within exosomes secreted from the glycolytic EDL, TA, gastrocnemius, and quadriceps normalized to the corresponding muscle weight were decreased to minimal levels after the age of 6 weeks (Figures 9A,C–E). In contrast, the exosomal levels of miR-206 released by soleus normalized to its weight were higher than the levels secreted by the other muscle tissues, indicating again its muscle fiber type specificity (Figure 9B). Spearman correlation analysis showed a positive correlation between the secreted miR-1, miR-133a, and miR-133b levels (rho > 063; *p* < 0.05) (Figures 9F–J). miR-206 exosomal levels, however, were found to be positively correlated with the other three myomiRs only in soleus-derived exosomes (rho > 0.66; *p* < 0.05) (Figure 9G).

### Endogenous MyomiR Levels Across Age Correlate to the Muscle-Derived Exosomal Levels

**FIGURE 10:**
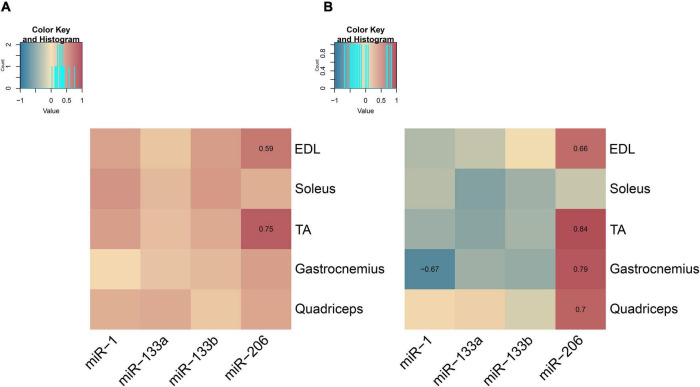
Correlation maps associating endogenous and muscle-derived exosomal myomiR levels for the five skeletal muscles. Correlation analysis of the endogenous and muscle-derived exosomal myomiR levels was performed using the Spearman method. **(A)** Association of endogenous and muscle-derived fold change levels of the four myomiRs showed a trend toward a positive correlation between them. Endogenous and muscle-derived exosomal myomiR levels relative to their levels at 2 weeks followed a similar profile across age for all five skeletal muscles. Both endogenous and muscle-derived exosomal levels were found to increase with age for each skeletal muscle of interest. **(B)** Correlation of the normalized endogenous and muscle-derived exosomal miR-1, miR-133a, and miR-133b levels over the corresponding muscle weight was found to be slightly negative. miR-206 endogenous levels per muscle weight were found to be positively correlated with its muscle-derived exosomal levels for all the glycolytic muscles, EDL, TA, gastrocnemius, and quadriceps. For the oxidative muscle soleus, no correlation was observed between miR-206 endogenous and muscle-derived exosomal levels normalized to its weight. Correlation (rho) values are depicted in the squares and those close to +1 (red) denote a strong positive correlation, and values close to –1 (blue) a strong negative correlation. Correlation values with no significance do not show their value. *p* < 0.05.

Exosomes are known to be involved in cell-to-cell and tissue-to-tissue crosstalk, with the majority of the studies focusing on cancerous cell lines or tumors (Caby et al., 2005; Lima and Möller, 2018). We have recently reported that muscle-derived exosomes may have an impact on local skeletal muscle communication in wild-type mice (Mytidou et al., 2021). In this study, we further investigated the association of muscle-derived exosomes to skeletal muscle endogenous myomiR expression during the first year of life in wild-type mice. The correlation between the endogenous and the muscle-derived exosomal myomiR profiles during muscle growth was assessed by employing correlation data analysis using the Spearman method. The fold change of the myomiR levels over the 2-week levels was initially analyzed. The endogenous and muscle-derived myomiR levels were found to be positively correlated for all examined skeletal muscles (Figure 10A and Supplementary Tables 1, 2). Interestingly, miR-206 presented two of the highest significantly positive correlation coefficients, for EDL and TA muscles (rho > 0.59; *p* < 0.05), implying a positive association between the endogenous fold change expression levels and its secreted levels, for both tissues (Figure 10A).

The normalized myomiR levels over muscle weights were next analyzed revealing a tendency toward negative correlation between the endogenous and secreted miR-1, miR-133a, and miR-133b levels for the majority of the investigated muscles (Figure 10B and Supplementary Tables 3, 4). The most prominent negative correlation was detected between the endogenous and secreted miR-1 levels in TA muscle (rho < −0.67; *p* < 0.05) (Figure 10B and Supplementary Tables 3, 4). By contrast, the endogenous levels of miR-206 normalized to muscle weight were found to be strongly positively correlated with the corresponding secreted miR-206 levels (rho > 0.66; *p* < 0.05) for the four glycolytic skeletal muscles, EDL, TA, gastrocnemius, and quadriceps (Figure 10B and Supplementary Tables 3, 4). However, the profile of endogenous miR-206 expression levels in soleus normalized over its weight across age displayed a lack of correlation with the soleus-secreted miR-206 normalized profile (Figure 10B).

### Circulating Exosomal MyomiR Levels in Serum Are Independent to the Age of the Mouse

It has been reported that under disease conditions, exosomal myomiRs are elevated in the blood circulation of muscular dystrophy patients compared to healthy controls (Koutsoulidou et al., 2015, 2017). Furthermore, recently exosomal myomiRs were detected in the serum of wild-type mice and suggested to be directly released by skeletal muscle tissues (Mytidou et al., 2021). There is no evidence, however, regarding exosomal myomiR levels in the serum of mice of different ages and their correlation to the endogenous and skeletal muscle-secreted levels. Exosomes circulating in the serum of wild-type male mice from 2 to 52 weeks of age were therefore next isolated and verified using scanning electron microscopy images, TRPS analysis and western blotting (Supplementary Figures 3A–C).

Following the successful identification of exosomes, the levels of encapsulated myomiRs were analyzed. Serum exosomes were found to be enriched in miR-133a and miR-133b levels, while miR-1 and miR-206 levels were low for all ages under investigation (Supplementary Figure 3D). Relative quantification of the four myomiRs encapsulated in serum exosomes was performed using the cel-miR-39 control. Furthermore, 2-week-old mice were determined to have the highest serum exosomal miR-133a, miR-133b, and miR-206 levels compared to older mice (Supplementary Figures 3D,E). This finding is possibly attributed to the maternal milk intake that takes place regularly at this early stage of life in mice. It is worth noting that for some of the studied ages exosomal miR-1 levels in serum presented over onefold increase compared to the 2 weeks, which is in line with the exosomal miR-1 profile secreted from skeletal muscles (Figures 8A–E and Supplementary Figure 3E). Spearman correlation analysis showed that the four exosomal myomiR levels in serum are positively correlated between them across age (rho > 0.58; *p* < 0.05) (Supplementary Figure 3F). Further association analysis of the serum exosomal myomiR fold change levels with the corresponding muscle-derived exosomal and endogenous myomiR levels unveiled the lack of a strong correlation between them (Supplementary Figure 4).

## Discussion

Myogenesis is a complex, tightly coordinated process, finely tuned by MRFs and miRNAs, which occurs before and after birth, to establish proper function of skeletal muscle (Sabourin and Rudnicki, 2000; Parker et al., 2003). A group of miRNAs, termed as myomiRs, was found to be expressed in a skeletal muscle specific manner and involved in the development and maintenance of skeletal muscle (McCarthy, 2008; Townley-Tilson et al., 2010; Horak et al., 2016). miR-1, miR-133a, miR-133b, and miR-206, are four myomiRs intensively studied for their crucial contribution in muscle homeostasis during embryogenesis and aged muscle, in *in vitro* and *in vivo* studies (Koutsoulidou et al., 2011; McGregor et al., 2014; Koutalianos et al., 2015). Furthermore, research has been carried out focusing on myomiR expression levels during muscle regeneration after exercise or injury. Specifically, after physical training or injury, quiescent satellite cells were identified to transit to an active cell cycle state, during which they proliferate and differentiate thus generating new muscle fibers (Ultimo et al., 2018; Iannone et al., 2020). Moreover, it was reported that the levels of the four myomiRs encapsulated within exosomes are elevated in the bloodstream of muscular dystrophy patients compared to healthy individuals (Cacchiarelli et al., 2011; Zaharieva et al., 2013; Koutsoulidou et al., 2017). The origin and fate of circulating myomiRs in the bloodstream still remain elusive, albeit their relation to the progression of muscular diseases has been determined (Zaharieva et al., 2013; Quattrocelli and Sampaolesi, 2015; Koutsoulidou et al., 2017). These observations indicated a possible mechanism employed by skeletal muscles to package myomiRs within exosomes and release them into the extracellular environment under disease conditions in humans.

To date, published data lack evidence regarding the four myomiR differential expression and function during developmental phases characterized by acute muscle growth, in the first year of life in animal models (Williams et al., 2009; Hamrick et al., 2010; Di Filippo et al., 2016; Vechetti et al., 2019). In the current longitudinal study, we examined the endogenous and muscle-derived exosomal levels of four myomiRs, miR-1, miR-133a, miR-133b, and miR-206, in thirteen different time-points across age, ranging from the early postnatal stage (2 weeks) up to adulthood (52 weeks), in five skeletal muscles of wild-type male mice. The course of both, the endogenous expression and the exosomal sorting of the four myomiRs in skeletal muscles, was found to be strongly associated to the growth of skeletal muscles across age. Up to early adulthood (12 weeks) healthy skeletal muscles acquire strength and flexibility and their mass is greatly increasing, while in elderly population (over 2 years of age) the vital muscle properties are lost, a condition known as sarcopenia (Rivas and Fielding, 2012; Walston, 2012). Here, we provided novel insights regarding the gradual alterations of myomiRs endogenous expression and exosomal secretion from skeletal muscles during rapid muscle gain and their possible involvement in adult muscle homeostasis.

Throughout the early postnatal period and adolescence (up to 8 weeks), the endogenous levels of the three myomiRs, miR-1, miR-133a, and miR-133b, were found to increase with age as muscles grow before reaching a plateau, indicating their importance during the first months of life. The similar increasing patterns observed in the endogenous levels of the three myomiRs and the skeletal muscle weights, after the 4-week age, can be attributed to the increase of skeletal myofibers which accompanies skeletal muscle growth. More specifically, as muscles gain weight with age, the number of myofibers increases thus contributing to the endogenous expression of the myomiRs. In addition, the three myomiRs’ expression levels during adulthood relative to the 2-week age were found to present a three to fourfold increase for the glycolytic muscles (EDL, TA, gastrocnemius, and quadriceps), whereas for the oxidative muscle soleus there was a two to threefold increase of their expression. These results imply that glycolytic muscles require higher levels of the three myomiRs for a normal growth compared to the oxidative types. On the contrary, endogenous miR-206 expression levels were identified to decrease drastically reaching minimal levels during muscle growth in the investigated glycolytic muscles, whereas in soleus miR-206 endogenous levels were found to increase following the same pattern with the other three myomiRs. Soleus mainly consists of slow and fast oxidative muscle fibers, while the rest skeletal muscles under investigation are predominantly composed of fast glycolytic muscle fibers (Pette and Staron, 2000; Schiaffino and Reggiani, 2011). miR-206 has been reported to be expressed in a specific muscle fiber type manner, favoring for oxidative types I and IIA thus explaining the differences that we observed in soleus (van Rooij et al., 2009; Bjorkman et al., 2020). Our finding also indicates that the endogenous expression of miR-206 is not as essential as the other three myomiRs during the development of healthy glycolytic skeletal muscles. However, oxidative skeletal muscles, such as soleus, require similar expression levels for all four myomiRs, demonstrating that muscle-fiber type specificity of miR-206 is consistent for all ages up to adulthood. MyomiR expression is mainly regulated by the four MRFs, myogenic factor 5 (Myf5), myoblast determination (MyoD), myogenic factor 4 (Myf4; myogenin), and myogenic factor 6 (Myf6) throughout the multi-steps of myogenesis (Sweetman et al., 2008; Koutsoulidou et al., 2011). Other muscle-related transcription factors are also involved in the expression of the four myomiRs, including myocyte enhancer 2 (Mef2) (Liu et al., 2007). The constant myomiR expression in the five skeletal muscles during muscle development from 2 up to 52 weeks after birth, as described in our study, suggests that myogenic inducers are also expressed in a continuous manner. Subsequently, it is further supported that the function of myogenic regulators is required not only for the formation of skeletal muscles when there is acute gain of weight, but for their maintenance through adult life as well.

Complementary to the longitudinal study of the four myomiR expression in the five hindlimb skeletal muscles, further analysis showed that SRF and Cx43 are inversely correlated to the myomiR levels. In all examined muscles, SRF levels decreased with age and muscle growth, while miR-133a and miR-133b endogenous levels increased. High SRF levels in the first 2–4 weeks of life in mice indicates the crucial role of this transcription factor when muscles gain mass rapidly, compared to the older ages where SRF is produced at lower, but stable, amounts. SRF has been linked to early myogenesis and regulation of skeletal muscle growth and its ablation in the early stages of muscle development resulted in premature death in mice (Charvet et al., 2006). On the contrary, when SRF was conditionally deleted in post-mitotic myofibers in adult mice, it was revealed that SRF is not crucial for muscle maintenance but is vital for muscle regeneration after injury (Lahoute et al., 2008). Furthermore, repression of SRF ensures sufficient population of satellite cells and myoblasts allowing proper muscle maturation. Taken together with our findings, it is further implied that high levels of SRF are related to active myogenic processes and the formation of new myofibers, while lower levels contribute to muscle aging networks and muscle atrophy (Lahoute et al., 2008; Sakuma et al., 2008). Moreover, the gradual reduction observed in the SRF levels of our analysis stops after 6 weeks of age, which coincides with muscles stop gaining large amounts of weight. Cx43 levels were identified to be inversely correlated to the endogenous expression of miR-206. Consequently, Cx43 levels were detected to alter across age in a myofiber-specific manner; they increased with age in the glycolytic muscles EDL, TA, gastrocnemius, and quadriceps, and decreased in the oxidative muscle soleus. Elevated Cx43 production in glycolytic muscles has been linked to increased membrane permeability and denervation of myofibers and hence contributes to muscle atrophy (Rowan et al., 2012; Cea et al., 2013). Altered membrane permeability in skeletal muscles results in increased Ca^2+^ intracellular influx, metabolic impairment and in particular high rates of protein degradation. Furthermore, deletion of Cx43 prevented muscle impairment in animal models exhibiting muscle diseases (Fernández et al., 2020; Nouet et al., 2020). Based on our results and published research, the observed increased levels of Cx43 in the glycolytic muscles of wild-type mice during the adolescence and adulthood could be considered as an indication for the initial stages of muscle atrophy in older muscles.

Further analysis of myomiR expression data using the tissues’ weights revealed that in the early postnatal phase (2 weeks), the endogenous four myomiRs’ levels per muscle weight are higher compared to the older ages. These results further emphasize the crucial role of the four myomiRs in the skeletal muscles’ development during the first 2 weeks of life in wild-type mice. After 4 weeks of age, the endogenous myomiR levels per mg remained at a constant value, indicating that the increasing pattern of myomiR endogenous expression as mice grow older is analogous to the muscle’s growth, for each skeletal muscle individually. In addition, the two smaller skeletal muscles, EDL and soleus, demonstrated higher myomiR levels per mg, for all investigated ages, compared to the other heavier tissues, implying that smaller muscles may have a distinct functional role in the muscular system which requires elevated amounts of myomiRs per mg. For instance, the elevated myomiR abundance in the small muscles could initiate myogenic networks at a higher rate, inducing different or complementary muscle functions to the bigger muscles. In particular, both EDL and soleus, belong to different muscle compartments, acting synergistically with the other larger proximal muscles, TA and gastrocnemius, respectively (Meijer et al., 2007; Stutzig and Siebert, 2015). Muscle synergies generate higher forces than single muscle activity and the smaller muscles compensate for the bigger muscles in terms of intense activity to retain a given movement (Stutzig and Siebert, 2015; Reinhardt et al., 2016). However, the underlying mechanism that supports the synergistic muscles still remains elusive. Our results imply that higher endogenous myomiR levels expressed in the smaller muscles may contribute to the synergistic model. Further research, however, is required to determine any association between myomiR expression and muscle synergies.

The exosomal secretion of myomiRs directly from the skeletal muscles under investigation was also studied. Exosomal packaging and release of the three myomiRs miR-1, miR-133a, and miR-133b from the five skeletal muscle tissues were found to increase with age during the first year of life in mice, in parallel with the endogenous myomiR levels. However, it is worth noting that the observed increase of the muscle-derived exosomal myomiR levels compared to the 2-week timepoint is steeper than the endogenous fold change. Exosomal myomiR levels secreted from the five skeletal muscles presented an increase of at least 5 times the 2-week levels, whereas the corresponding change of the endogenous levels was around 4 times. These increases were observed in the first 6 weeks of life, when mice enter adolescence. The observed fold change of the endogenous myomiR expression was similar to the increase in muscle weight, indicating an association between the myomiR expression and the size of the skeletal muscles. The differential myomiR encapsulation within muscle-derived exosomes, however, increased dramatically after the age of 4 weeks, compared to muscle growth. This finding implies that during the first 2–4 weeks of life, during which skeletal muscles grow rapidly and gain weight, the expressed myomiRs are retained within the tissue, further highlighting their important role for normal development of muscles. Moreover, considering that mice at the age of 2–4 weeks after birth (early postnatal stage) are static or with limited movements, while when they enter adolescence (6 weeks) they become more active and aggressive, it is further implied that exosomal release of the four myomiRs might be associated to the developmental stage of the mice. For example, skeletal muscles of older active mice may communicate at a higher rate through the exosomal route with other near or distant tissues compared to the early weeks of life when mice perform constricted body movements. Particularly, miR-1 revealed the biggest increase of its encapsulated levels within muscle-derived exosomes compared to 2 weeks age, implying that miR-1 is selectively secreted at high levels from skeletal muscles either to reach neighboring or more distant tissues, such as the cardiac muscle or the skeletal muscles of the forelimbs. In contrast, 2–4 weeks after birth, miR-1 expression levels were very low suggesting that miR-1 may be more essential during early postnatal myogenesis; thus, the exosomal miR-1 secretion was found to be restricted during this developmental phase. Moreover, muscle-derived exosomal miR-206 levels followed a different pattern compared to its endogenous levels relative to 2 weeks age. Exosomes released from EDL, TA, gastrocnemius, and quadriceps were found to encapsulate high miR-206 levels (up to 5 times compared to 2 weeks levels) during the first 6 weeks of life. Considering that the endogenous levels of miR-206 for the same skeletal muscles were found to decrease after birth, its redundancy within glycolytic muscles is further suggested. Nonetheless, miR-206 abundance within the glycolytic muscle-derived exosomes was identified to decrease after 6 weeks of age, along with its endogenous levels. By contrast, soleus-derived exosomes were found to encapsulate increasing miR-206 levels, compared to 2-week levels, until 6 weeks of age, before reaching a stable pattern over the course of time. This observation is in agreement with the miR-206 endogenous fold change trend, which further shows the myofiber specificity of this myomiR for the oxidative muscle soleus across age (van Rooij et al., 2009; Bjorkman et al., 2020).

In addition to the fold change of the muscle-derived exosomal myomiR levels over the 2-week levels, we also normalized the levels of myomiRs to the skeletal muscle weights. Our normalized data revealed that skeletal muscles tend to release less myomiR levels within exosomes per mg, during the early postnatal stage (2–4 weeks). This observation is the opposite of what it was presented for the normalized endogenous data, which revealed a high myomiR concentration for the same age groups. Combining the exosomal and endogenous myomiR profiles normalized over muscle weights, it is demonstrated that skeletal muscles during the first 4 weeks of life reduce the secretion of myomiR endogenous abundances, highlighting their vital involvement in proper muscle development at that period of time. After 4 weeks of age, skeletal muscles were determined to secrete higher exosomal levels of miR-1, miR-133a, and miR-133b per mg compared to 2 weeks. miR-206 levels were found to decrease within exosomes derived from the EDL, TA, gastrocnemius, and quadriceps muscles which is consistent with the normalized endogenous myomiR levels per mg. The soleus-derived exosomal levels of miR-206 were almost constant after the 4-week age.

We further assessed the correlation of the endogenous myomiR levels to the corresponding muscle-derived exosomal levels using the Spearman method. As expected, we observed a positive correlation between the endogenous and muscle-derived exosomal profiles, regarding the fold changes of the myomiR levels when compared to the 2-week levels. Both, endogenous expression and exosomal secretion of the four myomiR levels, presented a similar course during the first year of life in wild-type male mice. As healthy mice were growing with time, the endogenous and exosomal miR-1, miR-133a, and miR-133b levels were increasing and then they reached a plateau phase. Endogenous miR-206 levels were found to decrease right after birth during acute muscle gain, while exosomal miR-206 levels were identified to increase during the first 4 weeks and then decline in EDL, TA, gastrocnemius, and quadriceps, indicating that this myomiR is not important for the development of glycolytic skeletal muscles. By contrast, when associating the normalized values over the skeletal muscle weights, the endogenous and muscle-derived exosomal profiles for miR-1, miR-133a, and miR-133b were assessed as negatively correlated. During the early postnatal phase (2–4 weeks) the endogenous three myomiR levels per muscle weight were upregulated compared to the older ages, whereas during the same time period, muscle-derived exosomal myomiR levels per muscle weight were lower relative to the older ages. This negative correlation suggests that skeletal muscles express high myomiR concentrations but limit their exit to the extracellular environment, possibly to maintain a healthy development. Another reason supporting the negative correlation could be that other tissues may contribute to the increased endogenous myomiR levels within the skeletal muscles via myomiR delivery through the exosomal tissue crosstalk pathway or transportation by proteins.

Finally, analysis of the exosomal myomiR levels circulating in the bloodstream of wild-type male mice revealed that 2-week-old mice present upregulated levels of the four myomiRs. This finding combined with our result that 2-week skeletal muscles secrete exosomes with very low myomiR levels, could be associated to the intake of milk exosomes through breastfeeding (Rani et al., 2017; Manca et al., 2018; van Herwijnen et al., 2018). It is well established that milk was evolved to nourish and support the normal development of the new-borns. This is achieved by the milk’s rich molecular composition, which provides a wide range of bioactive molecules encapsulated or not within extracellular vesicles (Andreas et al., 2015; Sakaguchi et al., 2018). Therefore, the elevated serum exosomal myomiR levels observed in the early postnatal stage (2 weeks old), further emphasize their importance in this period in mice, during which significant changes are observed in the skeletal muscle development. Nonetheless, the exact origin of the exosomal myomiRs circulating in the blood circulation of mice remains to be elucidated.

In summary, this is the first longitudinal study of differential expression and secretion levels of the four myomiRs for five skeletal muscles of wild-type male mice, during their first year of life. Furthermore, we assessed the correlation between the expressed and secreted myomiR abundances. Our results suggest that the three myomiRs miR-1, miR-133a, and miR-133b are essential for proper adult skeletal muscle development and homeostasis, especially during the first weeks of life for all the examined skeletal muscles. In particular, the two small skeletal muscles, EDL and soleus, were found to express high levels of the three myomiRs per mg, whereas they release relatively low levels of these myomiRs within exosomes. This observation could be associated to their synergistic functions supporting other bigger muscles of the same compartment. miR-206 levels were found to be increased only within soleus and soleus-derived exosomes, maintaining its muscle fiber specificity for healthy mice up to 1 year old of age. After the early postnatal phase and adolescence (8–12 weeks), the myomiR expression and secretion remained relatively constant, a possible requirement for the continuous normal muscle maintenance. Collectively, our data analysis provided novel evidence regarding the development and function of skeletal muscles for the first year of life in mice. All these new insights could be employed for the in-depth understanding of healthy postnatal myogenesis and be used in future studies with disease animal models, especially in the case of muscular diseases, since molecularly and phenotypically they are related to premature aging and muscle wasting.

## Data Availability Statement

The original contributions presented in the study are included in the article/Supplementary Material, further inquiries can be directed to the corresponding author/s.

## Ethics Statement

The animal study was reviewed and approved by the European Directive 2010/63/EU and Cyprus Legislation, Laws 1994–2013.

## Author Contributions

LP conceived the study and coordinated the study. LP and AK designed the study. CM performed most of the research and wrote the manuscript. AA, MP, and KK performed scanning electron microscopy and TRPS experiments. GS and MZ performed the correlation analysis. LP, AK, and CM analyzed and interpreted the data. All authors read and approved the final manuscript.

## Conflict of Interest

MP was employed by the company Theramir Ltd. The remaining authors declare that the research was conducted in the absence of any commercial or financial relationships that could be construed as a potential conflict of interest.

## Publisher’s Note

All claims expressed in this article are solely those of the authors and do not necessarily represent those of their affiliated organizations, or those of the publisher, the editors and the reviewers. Any product that may be evaluated in this article, or claim that may be made by its manufacturer, is not guaranteed or endorsed by the publisher.
