# Supplementary material for: Age-Related Exosomal and Endogenous Expression Patterns of miR-1, miR-133a, miR-133b, and miR-206 in Skeletal Muscles
Source: Front Physiol. 2021 Nov 18;12:708278. doi: 10.3389/fphys.2021.708278 (PMC8637414; doi:10.3389/fphys.2021.708278)
Supplement: Supplementary file 1 [file Table_1.pdf]

Supplementary Table 1: Correlation analysis among the endogenous and muscle-derived fold change values for the four myomiRs.

	**miR-1**	**miR-133a**	**miR-133b**	**miR-206**
**EDL**	0.3407	0.1429	0.3791	**0.5879**
**Soleus**	0.4231	0.2033	0.3901	0.2692
**TA**	0.3626	0.1813	0.3022	**0.7527**
**Gastrocnemius**	0.0220	0.1593	0.2088	0.3626
**Quadriceps**	0.2692	0.3022	0.1264	0.3297

Correlation values were calculated with the Spearman method and are shown for each myomiR per muscle. Correlation values with p-values less than 0.05 are in bold text (see Table S2 for equivalent p-values).
