# Supplementary material for: Age-Related Exosomal and Endogenous Expression Patterns of miR-1, miR-133a, miR-133b, and miR-206 in Skeletal Muscles
Source: Front Physiol. 2021 Nov 18;12:708278. doi: 10.3389/fphys.2021.708278 (PMC8637414; doi:10.3389/fphys.2021.708278)
Supplement: Supplementary file 2 [file Table_2.pdf]

Supplementary Table 2: P-values for the correlation analysis among the endogenous and muscle-derived fold change values.

	**miR-1**	**miR-133a**	**miR-133b**	**miR-206**
**EDL**	0.2547	0.6415	0.2014	**0.0346**
**Soleus**	0.1497	0.5053	0.1876	0.3737
**TA**	0.2233	0.5533	0.3156	**0.0030**
**Gastrocnemius**	0.9432	0.6031	0.4936	0.2233
**Quadriceps**	0.3737	0.3156	0.6808	0.2713

The p-values are shown for each myomiR per muscle. P-values less than 0.05 are in bold text.
