# Supplementary material for: Age-Related Exosomal and Endogenous Expression Patterns of miR-1, miR-133a, miR-133b, and miR-206 in Skeletal Muscles
Source: Front Physiol. 2021 Nov 18;12:708278. doi: 10.3389/fphys.2021.708278 (PMC8637414; doi:10.3389/fphys.2021.708278)
Supplement: Supplementary file 3 [file Table_3.pdf]

Supplementary Table 3: Correlation analysis among the endogenous and muscle-derived normalised relative quantification values for the four myomiRs.

	**miR-1**	**miR-133a**	**miR-133b**	**miR-206**
**EDL**	-0.2802	-0.2088	-0.0110	**0.6648**
**Soleus**	-0.2582	-0.4890	-0.3571	-0.1868
**TA**	-0.3736	-0.4505	-0.3352	**0.8352**
**Gastrocnemius**	**-0.6703**	-0.3626	-0.4066	**0.7857**
**Quadriceps**	0.0330	0.0824	-0.1319	**0.7033**

Correlation values were calculated with the Spearman method and are shown for each myomiR per muscle. Correlation values with p-values less than 0.05 are in bold text (see Table S4 for equivalent p-values).
