# Supplementary material for: Age-Related Exosomal and Endogenous Expression Patterns of miR-1, miR-133a, miR-133b, and miR-206 in Skeletal Muscles
Source: Front Physiol. 2021 Nov 18;12:708278. doi: 10.3389/fphys.2021.708278 (PMC8637414; doi:10.3389/fphys.2021.708278)
Supplement: Supplementary file 4 [file Table_4.pdf]

Supplementary Table 4: P-values for the correlation analysis among the endogenous and muscle-derived normalised relative quantification values.

	**miR-1**	**miR-133a**	**miR-133b**	**miR-206**
**EDL**	0.3538	0.4936	0.9716	**0.0132**
**Soleus**	0.3943	0.0899	0.2309	0.5411
**TA**	0.2086	0.1223	0.2629	**0.0004**
**Gastrocnemius**	**0.0122**	0.2233	0.1680	**0.0015**
**Quadriceps**	0.9149	0.7890	0.6676	**0.0073**

The p-values are shown for each myomiR per muscle. P-values less than 0.05 are in bold text.
