# Supplementary figures and images for: Age-Related Exosomal and Endogenous Expression Patterns of miR-1, miR-133a, miR-133b, and miR-206 in Skeletal Muscles
Source: Front Physiol. 2021 Nov 18;12:708278. doi: 10.3389/fphys.2021.708278 (PMC8637414; doi:10.3389/fphys.2021.708278)

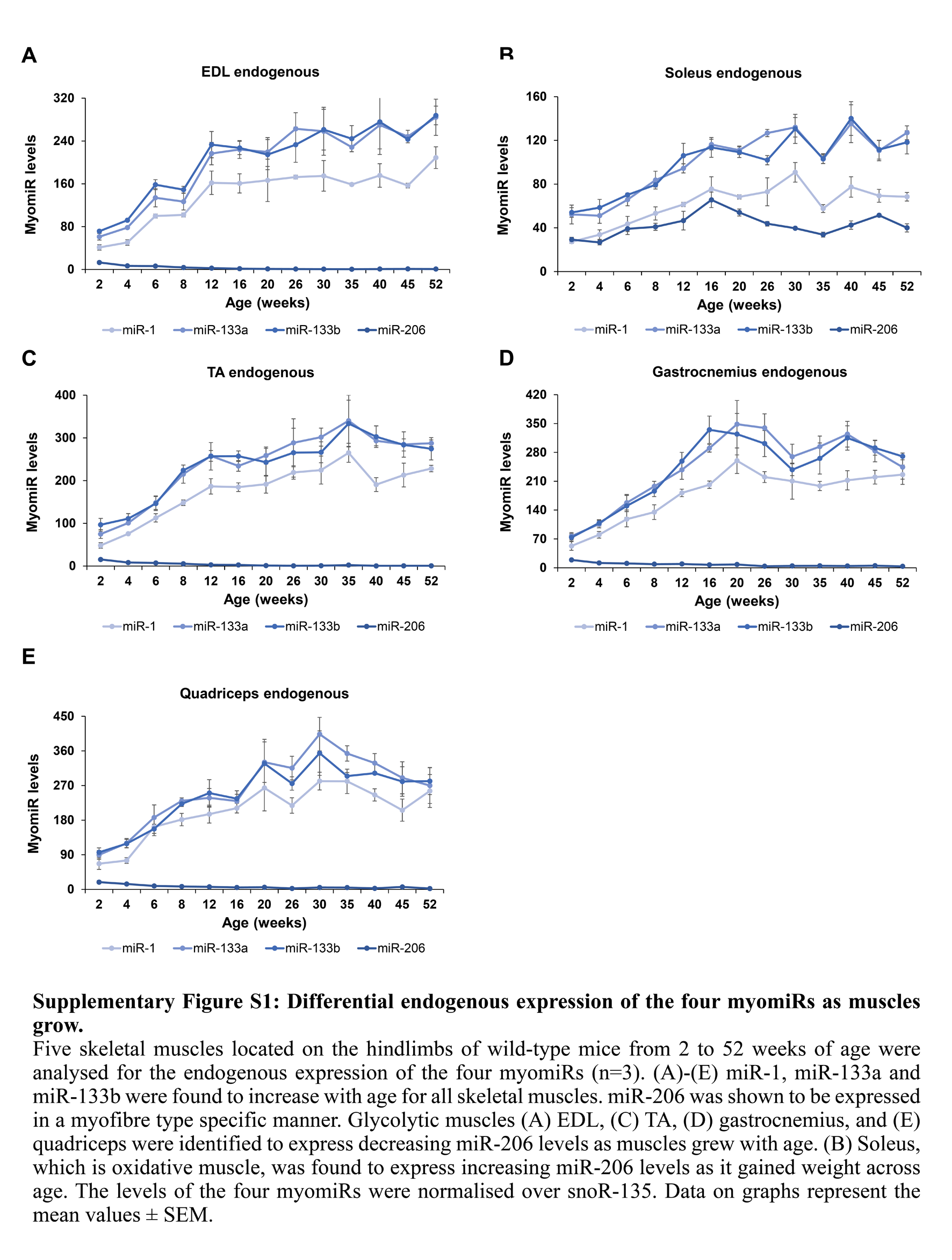

Supplement: Supplementary file 5 [file Image_1.TIF]

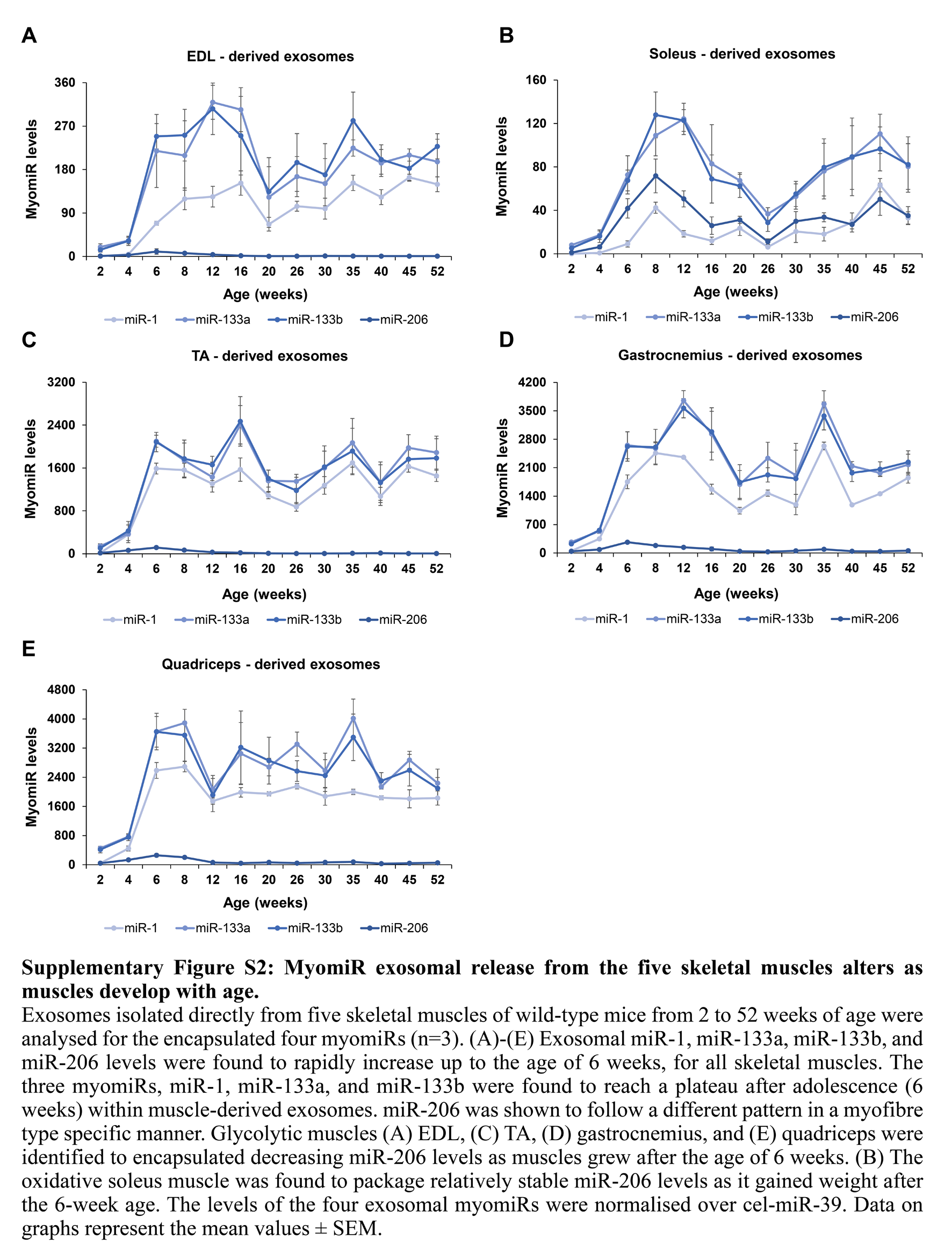

Supplement: Supplementary file 6 [file Image_2.TIF]

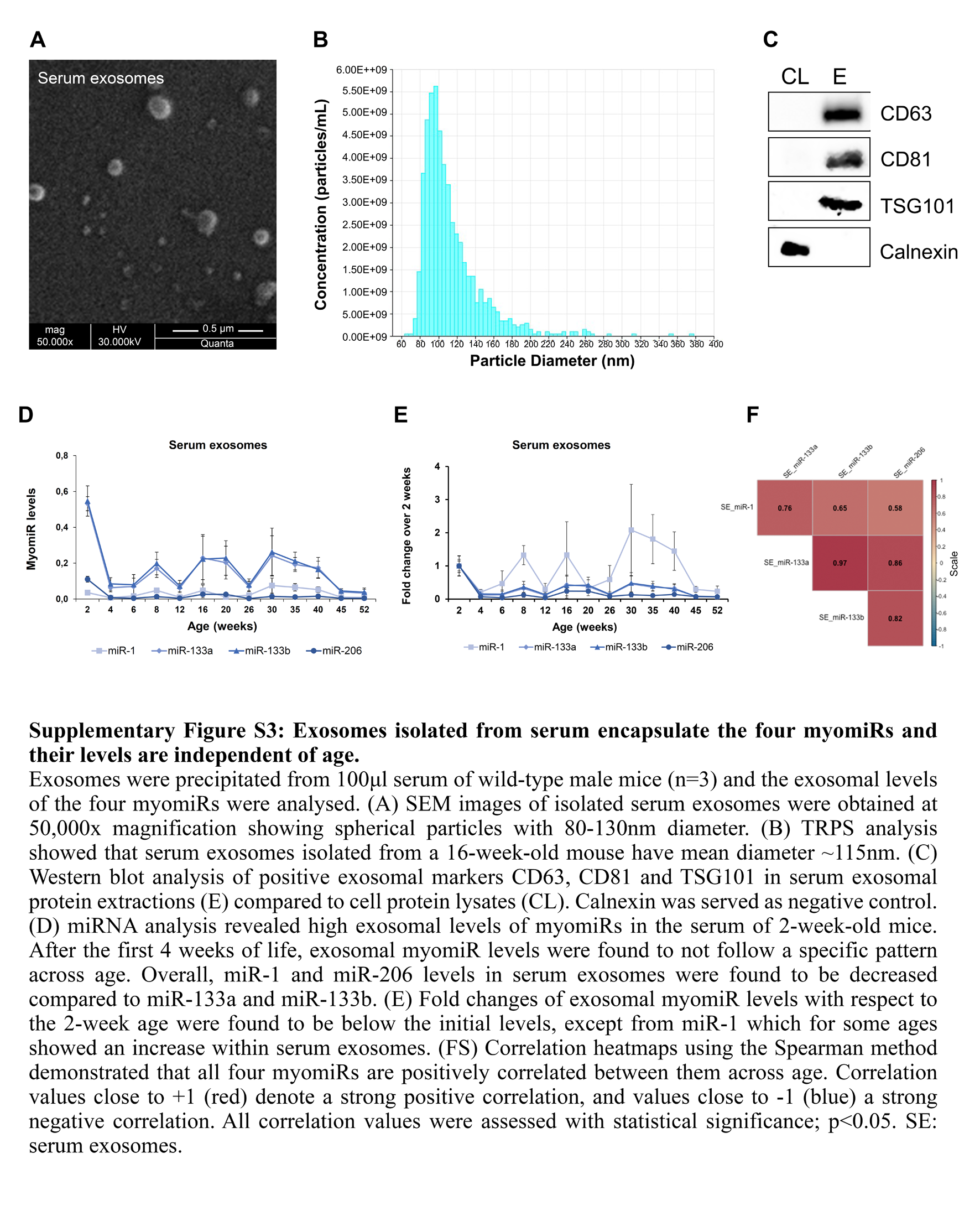

Supplement: Supplementary file 7 [file Image_3.TIF]

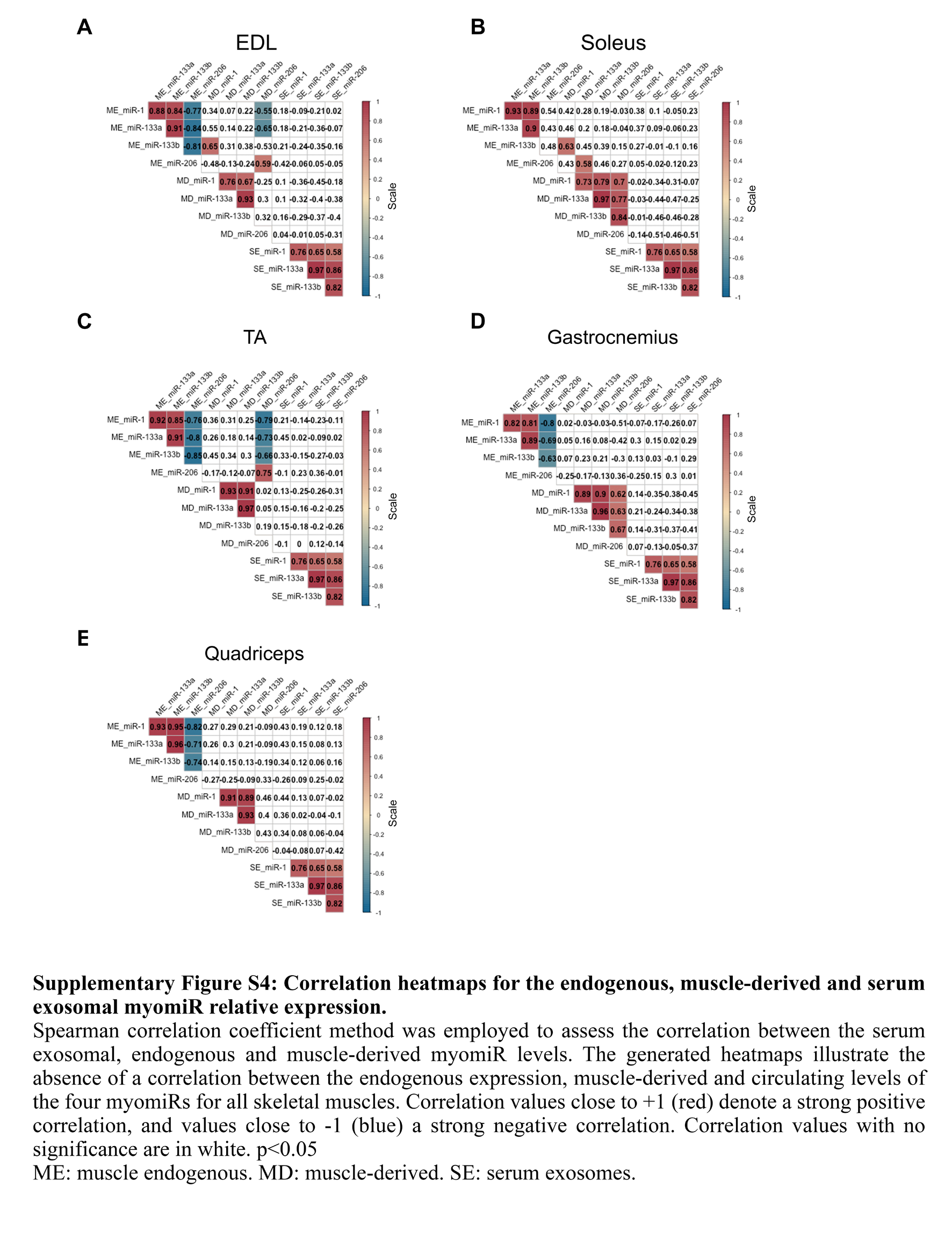

Supplement: Supplementary file 8 [file Image_4.TIF]
